# *Syzygium cumini* inhibits growth and induces apoptosis in cervical cancer cell lines: a primary study

**DOI:** 10.3332/ecancer.2008.83

**Published:** 2008-08-21

**Authors:** D Barh, G Viswanathan

**Affiliations:** Cancer Research Group, IHMA, Tamil Nadu 613006, India

## Abstract

Cervical cancer is common among women in the Indian subcontinent and the incidences and death rates are gradually increasing over the years. Several dietary phytochemicals have been reported to have growth inhibitory and apoptotic effect on HeLa and other cervical cell lines. In this study, using Hoechst 33342 staining, MTT, Annexin V-FLUOS/PI and TUNEL assays we demonstrated that ***Syzygium cumini*** extract inhibits the growth and induces apoptosis in HeLa and SiHa cervical cancer cell lines in a dose- and time-dependent manner. The phytochemical, its mode of action and safety issues are yet to be determined.

## Introduction

Cervical cancer is the second most common cancer in women worldwide [1] with about a half-million new cases diagnosed each year [2]. According to the Indian National Cancer Registry Programme of the ICMR (1997), cervical cancer is the most common cancer in Indian women, followed by breast, oesophagus, ovary and stomach (Table 1) and the incidence is increasing, with an estimated rate of 100,000 new cases per year [3]. Standard treatment for cervical cancer, that is surgery along with taxol chemotherapy, often results in severe myelotoxicity and neurotoxicity [4,5]. On the contrary, the strategy of targeted apoptosis to neoplastic cells [6] using dietary phytochemicals having immunostimulative, antioxidant, anti-neoplastic, apoptotic and other physiological benefits are showing promising results in ***in vitro*** studies for several cancers.

Apoptotic and growth inhibitory effects of curcumin [7–10], aloe emodin [11], resveratrol [12–18], retinoic acid [19–22], lycopene [23,24] and the tea polyphenol EGCG [25–28] in HeLa and other cervical cancer cell lines is well documented.

In this study, we evaluated the potential growth inhibitory and apoptotic effects of ***Syzygium cumini*** extract on two cervical cancer cell lines (HeLa and SiHa).

## Materials and methods

### Syzygium cumini *extract*

Crude extracts were isolated from wild-type partially ripe fruit skin along with the outermost layer of the berry, and then serial dilutions of the extract from 100% to 10% were made using PBS for MTT assays. Methanol extracts were prepared for other experiments by blending 5 g of skin with 50 ml of methanol at 4°C. After incubation at 37°C for 15 minutes, the extract was centrifuged at 3000 rpm for 10 minutes at 4°C. The supernatant was filtered, and the filtrate was vacuum dried and stored at 4°C. For use the dried extract was dissolved in DMSO and diluted with culture medium to a final concentration of 80% (v/v).

### Cell culture

Human cervical carcinoma cell lines HeLa (HPV-18 positive) and SiHa (HPV-16 positive) were cultured in DMEM medium (supplemented with 10% FBS, 2 mM glutamine, 100 IU/ml penicillin, and 100 μg/ml streptomycin) in 96-well plates at 37°C with 5% CO_2_ and air humidity 95%. Exponentially, growing cells were used for experiments.

A haemocytometer was used for cell counting using the formula:

Average count/4 × 104 × 10 (dilution factor) = 1.05 × 106 cells/ml

Total number of cells = 1.05 × 106 cells/ml × 5 ml = 5.25 × 107 cells

### MTT assay

The cytotoxic effect of ***Syzygium cumini*** crude extract on HeLa and SiHa cells was initially determined by MTT assay. In a 96-well plate, 0.25 ml cultures were added to 0.1 ml medium and incubated for two hours. Fifty μl of 100% or 10% extract were added to specific test wells; 50 μl PBS was used as a control. After 24 hours of culture 100 μl of MTT solution (5.0 mg/ml) was added to each well and left for 90 minutes. Culture were then washed with 0.1 ml of 10% SDS in a low-speed shaker for 3 hours and then centrifuged at 100 rpm for 5 minutes. The absorbance of 0.9 ml of cell supernatant was read at 570 nm against a blank (medium alone). The growth inhibition was measured using the following formula:

Percentage inhibition = [A570(control) − A570 (sample)/A570(control)] × 100.

**Figure 1:**
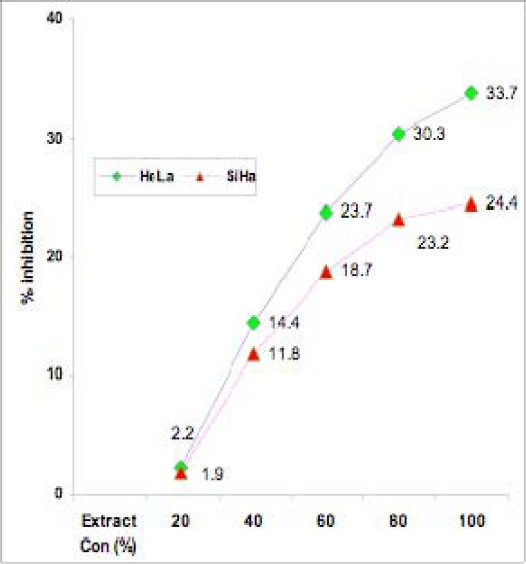
Effect of different concentrations of *Syzygium cumini* crude extract on growth inhibition of HeLa and SiHa cells, as determined by MTT assay

The average growth inhibition percentages of triplicates are represented in Figure 1.

Morphological features of apoptosis were evaluated with Hoechst 33342 staining, TUNEL (terminal deoxynucleotidyl transferase (TdT)-catalysed dUTP-nick end labelling), and Annexin V-FLUOS/PI (Propidium Imidone) binding assays using phase contrast and fluorescence microscopy.

### Hoechst 33342 staining

HeLa and SiHa cells seeded in chamber slides overnight were treated with 80% (v/v) ***Syzygium cumini*** extract. After 24, 36 and 48 hours of treatment, the medium was removed and the cells were washed twice with PBS. Cells were then fixed in 3% paraformaldehyde for 30 minutes, washed and stained with Hoechst 33342 (10 μg/ml) (Sigma) at 37°C for 30 minutes in the dark. After three washes with PBS, mounted slides were viewed under a fluorescence microscope at 450–490 nm. The percentage of apoptotic cells was determined by cell counting, taking the mean count of five microscopic fields (Figure 3A).

### Annexin V-FLUOS/PI assay

Annexin V-FLUOS/PI Staining Kit (Roche diagnostic) was used to determine membrane morphology of treated cells according to the manufacturer’s protocol. Briefly, cells were washed with PBS and resuspended in binding buffer (2.5 mM CaCl2, 140 mM NaCl, 10 mM Hepes-NaOH, pH 7.4) followed by the addition of fluorescence conjugated Annexin-V (1 μg/ml) and 5 μg/ml PI and then a 30 minute incubation in the dark. Cells were observed under a fluorescence microscope at 488 nm.

### TUNEL assay

TUNEL assay was performed using an ***in situ*** Cell Death Detection Kit (Roche diagnostic), following the manufacturer’s guidelines. In brief, cells were fixed with 4% paraformaldehyde and permeabilized (0.1% sodium citrate and 0.1% Triton X-100) after washing. Cells were then rewashed and incubated with the TUNEL assay reaction mixture for 1 hour at 37°C and 95% air humidity. Samples were then analysed under a fluorescence microscope at 488 nm.

## Results

A prominent growth inhibitory effect of ***Syzygium cumini*** extract on these two cervical cell lines was observed. Different dilutions of the extract exhibited growth inhibition in a dose-dependent manner in both cell lines tested with the MTT assay. While the 40% concentration of the extract exhibited 14.4% (HeLa) and 11.8% (SiHa) growth inhibition, the 80% concentrated extract showed 30.3% and 23.2% growth inhibition, respectively, in HeLa and SiHa cell lines. The 100% concentration did not show any significant effect over the 80% concentration. These results also indicate that the extract is more effective on HeLa than on SiHa cells (Figure 1).

**Figure 2:**
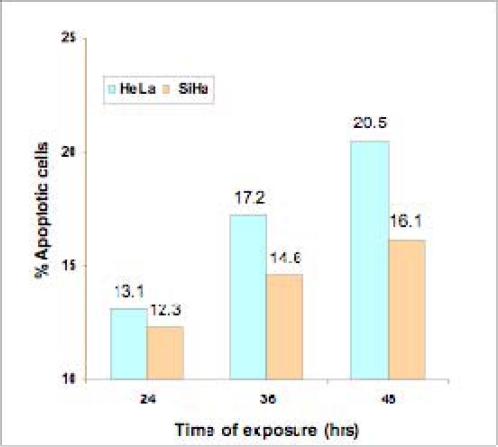
Effect of different exposure duration of *Syzygium cumini* methanolic extract on growth inhibition of HeLa and SiHa cells as determined by Hoechst 33342 staining

**Figure 3:**
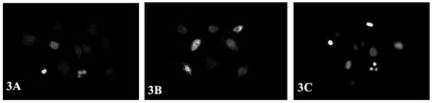
Effect of *Syzygium cumini* methanolic extract on growth inhibition of cervical cancer cell lines. (A) Hoechst 33342 staining for SiHa cells at 36 hours; (B) Annexin-V binding assay for HeLa cells at 48 hours; (C) TUNEL assay for SiHa cells at 48 hours.

Hoechst 33342 staining indicated condensed chromatin, the characteristic pattern of apoptosis, for both the cell lines under experiment. The apoptotic indices for these cell lines were calculated as mentioned earlier. It has been observed that a gradual increase of apoptotic index followed according to the treatment duration. Apoptotic cell counts were carried out at 24 hours (HeLa: 13.1%, SiHa: 12.3%), at 36 hours (HeLa: 17.2%, SiHa: 14.6%) and at 48 hours (HeLa: 20.5%, SiHa: 16.1%) (Figure 2). It has also been noticed that the methanolic extract was less effective as compared to the crude extract at the same concentration and the same exposure time. The result for SiHa is shown in (Figure 3A).

To distinguish the nuclear and membrane changes of the cells during apoptosis and also to investigate the necrotic cell death simultaneously, dual staining of cells with Annexin-V and PI was carried out with Annexin-V FLUOS/ PI staining. No PI staining was observed after 24 hours treatment, ruling out the possibility of necrosis, but fluorescence due to Annexin-V binding to phosphatidylserine on the outer cell membrane of the apoptotic cells was observed. Figure 3B represents the Annexin-V binding assay for HeLa cells at 48 hours.

The TUNEL assay was performed to detect the DNA strand breaks associated with apoptosis. The typical DNA strand breaks were detected in apoptotic cells after 24 hours treatment as strong fluorescence; these were absent in controls. Figure 3C) represents the TUNEL assay micrograph for SiHa cells at 48 hours.

## Discussion

Cervical cancer is the second cause of death from cancer in women worldwide, and the pathogenesis is well identified [29–31]. The treatment options generally followed are mainly surgery with or without adjuvant radiotherapy and chemotherapy [32–34], which often results in severe side effects.

Medicinal plants play a major role in folk medicine in several developing countries. The dietary bioactive phytochemicals, namely curcumin, resveratrol, emodin, retinoic acid, lycopene, EGCG, and indole-3-carbinol, are not only anti-cancerous, anti-proliferetive and apoptotic but also have antioxidant, anti-diabetic, anti-mutagenic and various other physiological benefits and so represent a potential alternative to conventional chemotherapy and radiotherapy for cervical cancer. The screening of medicinal plants for potential anti-cancer properties has increased greatly over the years.

***Syzygium cumini*** of the Myrtaceae family contains bergenin (an isocoumarin) [35], myricetin (a flavonol) and several polyphenols [36,37], tannins, http://www.herbs2000.com/h_menu/tannins.htm alkaloids (Jambosine), triterpenoids and volatile oils. Various parts of the plant are used for treatment of a variety of human abnormalities. The seed is able to lower blood glucose rapidly and so is effective in treating diabetes [38,39] and hyperinsulinemia [40]. Seed extracts are rich in phenols and show high-antioxidant activity [41]. It inhibits gastric ulceration [42], protects from gamma radiation [43], reduces the damage to brain tissue of diabetics [44] and also inhibits alpha-glucosidase activity [45]. The leaf extract has anti-fungal properties [46] and is anti-hyperglycemic and lowers blood glucose level in type 2 diabetes [47,48]. It reduces radiation-induced DNA damage in cultured human lymphocytes [49], can dissolve human gallbladder stones *in vitro* [50], protects from carbon tetrachloride-induced hepatotoxicity [51] and inhibits goatpox virus replication [52]. Its anti-allergic and anti-edematogenic effect is brought about by inhibiting histamine, serotonin, CCL11, IL-5 and mast cell degranulation [53]. Essential oils from the leaf have been reported to have anti-bacterial activity [54] and the bark has anti-inflammatory [55], gastroprotective [56] and anti-diarrhoeal [57] activities. The fruit skin has been reported to have antioxidant activity [58] and the fruit pulp an anti-hyperglycemic effect [59].

This study shows that extract of ***Syzygium cumini*** fruit skin along with the outermost layer of the berry inhibits growth and induces apoptosis in both HeLa and SiHa cervical cancer cell lines in a dose- and time-dependent manner. Whereas the crude extract exhibited, respectively, 33.7 % and 24.4% growth inhibition in HeLa and SiHa cells at its highest concentration (100%) in MTT assay, the methanolic extract (80% v/v) showed an apoptotic index of 20.5% and 16.1%, respectively, for these cell lines as determined by Hoechst 33342 staining. It has also been found that the crude extract is more effective in growth inhibition and apoptosis than the methanolic extract at its most effective concentration (80%). Annexin-V binding and TUNEL assays also confirm the apoptotic effect of the extract.

## Conclusion

In conclusion, the study confirms a dose- and time-dependent growth inhibitory and apoptotic effect of ***Syzygium cumini*** extract on the cervical cancer cell lines HeLa (HPV-18 positive) and SiHa (HPV-16 positive). ***Syzygium cumini*** fruit contains gallic acid, which has anti-adenoviral, anti-HIV, anti-peroxidant, anti-carcinomic, apoptotic and chemopreventive activities and is a topoisomerase-I-inhibitor (Dr Duke’s Phytochemical and Ethnobotanical Databases: http://www.ars-tgrin.gov/duke/). Though the results of this study indicate the growth inhibitory and apoptotic effect of the extract, the chemical component(s) responsible for these phenomena is/are yet to be precisely identified. Similarly, the anti-viral effect in respect to HPV-18 and HPV-16 is yet to be confirmed by further experiments. Moreover, further research is needed to identify the mode of action, efficacy and safety issues.

## Figures and Tables

**Table 1:**
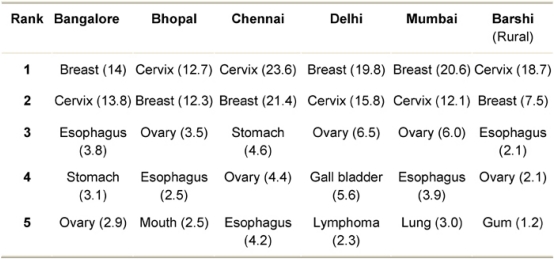
Leading cancers in population-based cancer registries under the National Cancer Registry Program of ICMR (1997) for women. Figures in parenthesis are crude incidence rates per 100,000
